# Phylogeographic study of Chinese seabuckthorn (*Hippophae rhamnoides* subsp. *sinensis* Rousi) reveals two distinct haplotype groups and multiple microrefugia on the Qinghai-Tibet Plateau

**DOI:** 10.1002/ece3.1295

**Published:** 2014-10-28

**Authors:** Hongfang Wang, Han Liu, Mingbo Yang, Lei Bao, Jianping Ge

**Affiliations:** State Key Laboratory of Earth Surface Processes and Resource Ecology & College of Life Sciences, Beijing Normal UniversityBeijing, 100875, China

**Keywords:** Analyses of molecular variance, beast, chloroplast intergenic fragments, demographic analysis, *Hippophae rhamnoides* subsp. *sinensis*, historical climate change

## Abstract

Historical climate change can shape the genetic pattern of a species. Studies on this phenomenon provide great advantage in predicting the response of species to current and future global climate change. Chinese seabuckthorn (*Hippophae rhamnoides* subsp. *sinensis*) is one of the most important cultivated plants in Northwest China. However, the subspecies history and the potential genetic resources within the subspecies range remain unclear. In this study, we utilized two intergenic chloroplast regions to characterize the spatial genetic distribution of the species. We found 19 haplotypes in total, 12 of which were unique to the Chinese seabuckthorn. The populations observed on the Qinghai-Tibet Plateau (QTP) consisted of most of the haplotypes, while in the northeast of the range of the subspecies, an area not on the QTP, only four haplotypes were detected. Our study also revealed two distinct haplotype groups of the subspecies with a sharp transition region located in the south of the Zoige Basin. 89.96% of the genetic variation located between the regions. Mismatch analysis indicated old expansions of these two haplotype groups, approximately around the early stage of Pleistocene. Additional morphological proofs from existing studies and habitat differentiation supported a long independent colonization history among the two regions. Potential adaptation probably occurred but needs more genome and morphology data in future. Chinese seabuckthorn have an older population expansion compared with subspecies in Europe. The lack of large land ice sheets and the heterogeneous landscape of the QTP could have provided extensive microrefugia for Chinese seabuckthorn during the glaciation period. Multiple localities sustaining high-frequency private haplotypes support this hypothesis. Our study gives clear insight into the distribution of genetic resources and the evolutionary history of Chinese seabuckthorn.

## Introduction

Global changes have occurred throughout the Earth's history and will continue to in the future. Thus, the response of species to these changes is a central issue for the study of biodiversity in the face of current global change (Hewitt 2000; Hampe and Petit 2005). Phylogeography provides an important tool to look into the history of species. Using genetic markers, phylogeography aims to find the processes underlying the geographic distribution of genealogical lineages (Avise et al. 1987; Avise 2000, 2004). Processes that possibly shape the genetic structure of the population include population demography, gene flow, genetic drift, and selection (Avise 2004). Combining phylogeography results with information from paleopalynology and related fields, it is possible to uncover the coupled history of species and global change. To date, phylogeography has yielded great success in many studies such as the history of plants (Petit et al. 2002; Londo et al. 2006; Artyukova et al. 2011; Sakaguchi et al. 2012), animals (Narita et al. 2007; Aurelle et al. 2011; Davison et al. 2011), and many diseases (Perkins 2001; Bargues et al. 2006).

*Hippophae rhamnoides* is a species widely distributed in Europe and Asia, with eight subspecies according to the distinguishable characteristics of fruits, flowers, and leaves (Rousi 1971; Lian and Chen 1991, 1996; Swenson and Bartish 2003). Phylogeny studies based on chloroplast and nuclear markers also suggest the monophyly of *H. rhamnoides* (Bartish et al. 2002; Sun et al. 2002). However, due to frequent hybridization between species or subspecies and great morphological variation within a range of species (Lian et al. 1997c; Aras et al. 2007; Du et al. 2008; Wang et al. 2008; Cheng et al. 2009), part of the taxonomic status of the species remains obscure. For example, *H. rhamnoides* subsp. *sinensis* (Chinese seabuckthorn) and *H. rhamnoides* subsp. *yunnanensis* have very close relationship, according to extant phylogeny studies (Bartish et al. 2002). The morphology differences of the two subspecies are small, most representing as leaves, shoots, and fruits. For instance, Chinese seabuckthorn has opposite or subopposite leaves, while subsp. *yunnanensis* has exclusively alternate leaves. The two subspecies ranges have some overlap on east of the QTP. Therefore, introgression or incomplete lineage sorting is possible to occur between the two subspecies. However, no population genetic study has been taken so far to investigate the taxonomic status of the two subspecies.

*Hippophae rhamnoides* subsp. *sinensis* (Chinese seabuckthorn) is a subspecies with the one of the largest distribution ranges. It distributes from Northeast to Southwest of China, where the elevation changes dramatically from a few hundred to thousands of meters. Chinese seabuckthorn has the longest cultivation and plantation history among the genus and plays a very important role in the economic development and environmental protection north of China (Li 2005). Abundant morphological variation has been described within the subspecies (Lian et al. 1997a; Huang 2003; Wu et al. 2007). It is critical to identify the genetic basis of these variations and uncover the evolution history of the subspecies. As the area of the plantation increases, the natural spatial genetic distribution is gradually blurred, which may ultimately eliminate the chance to understand the natural history of the subspecies in the near future.

Phylogeography studies of some subspecies of *H. rhamnoides* and related species suggest that most populations of the genus may have been restricted to the south range during the glacial period and expanded after glaciations. *Hippophae tibetana* had four distinct chloroplast haplotype groups spatially isolated from each other, which may indicate more than one refugia along the south and east edges of Qinghai-Tibetan Plateau (QTP) during the glacial periods (Wang et al. 2010; Jia et al. 2011). Using chloroplast and nuclear markers, Bartish et al. (2006) revealed a similar pattern of the four subspecies of *H. rhamnoides* in Europe and Asia Minor, with a diversity center in Southeastern Europe. Both studies showed a significant population expansion after glaciations. For Chinese seabuckthorn, genetic studies of the population using traditional nuclear genetic markers like ISSR and RAPD showed high genetic diversity and low genetic differentiation among populations (Sun et al. 2004; Zhang et al. 2006). However, due to limitation of the sample range and resolution of the molecular markers they used, no comprehensive history has been told for this subspecies.

Due to the wide distribution of Chinese seabuckthorn, the subspecies has grown in very different habitats across the range. The habitat located on the QTP consists of an area predominately at an altitude higher than 3000 m and experiences very abundant precipitation (more than 600 mm per year). The habitat not on the QTP is at lower altitude and experiences less precipitation. Morphological differences in fruits and branches have been described in these two kinds of habitats, suggesting that the subspecies may have originated along the edge of the QTP and subsequently expanded to the other parts (Lian et al. 1997a; Huang 2003). The edge of the QTP may have possibly supported relict populations during the glacial period, which has been proven by phylogeography studies of many species on the plateau (Zhang et al. 2005; Yang et al. 2008; Li et al. 2010; Qu et al. 2010; Wang et al. 2010). However, as there were no big and continuous ice sheets in China during the glacial periods, except some high mountain areas (Shi et al. 2006), we still lack concrete proof that all the populations of Chinese seabuckthorn had been restricted to the edge of the QTP and no refugia had existed in the other parts.

In this study, we used two chloroplast sequence fragments to characterize the genetic variation distribution across the range of Chinese seabuckthorn. We aimed to answer the following three questions:

1. What is the spatial genetic pattern of the subspecies? Do the differences of the habitats on the QTP and the other range region contribute to this pattern?
2. How did the subspecies survive the glacial period? Did multiple refugia exist both on the QTP and on the other range region, or was there only a single refugium?
3. As *H. rhamnoides* subsp. *yunnanensis* was the close relative of Chinese seabuckthorn, what is the genetic relationship between two close relatives *H. rhamnoides* subsp. *yunnanensis* and Chinese seabuckthorn?

## Materials and Methods

### Field sampling and sequencing

In 2005 and 2007, we collected the leaves from 19 populations of Chinese seabuckthorn, which covered the whole range of the subspecies (Table 1). Five to seven samples of each population and totally 109 samples were collected. The leaves were then stored in silica gel until DNA extraction. As clone reproduction exists in the subspecies, only adult individuals at least 20 meters apart from each other were sampled. To ensure we collected the right samples, photographs of most individuals were taken and rechecked against a reference.

**Table 1:** Sampled populations in this study. *N*, number of individuals sampled in each population; *T*_a_, annual mean temperature; *P*_a_, annual precipitation. Temperature and precipitation data were extracted from China environment raster datasets (http://159.226.111.42/pingtai/tupian/) in Arcmap

Code	Locations	Longitude	Latitude	*N*	Altitude (m)	*T*_a_ (°C)	*P*_a_ (mm)
*Hippophae rhamnoides* subsp. *sinensis*							
NeiM	Chifeng, Inner Mongolia	119.76	42.42	6	563	6.8	427.2
HbWC	Weichang, Hebei	117.69	41.98	5	904	5.3	405.2
HbSY	Weichang, Hebei	117.09	41.89	5	1396	2.4	432.8
SxYY	Youyu, Shanxi	112.86	40.08	5	1272	5.6	399.1
ShanX	Kelan, Shanxi	111.55	38.69	6	1472	5.9	438.8
SxQY	Qinyuan, Shanxi	112.07	36.52	5	1234	10.1	552.9
QingH	Xining, Qinghai	101.79	36.64	5	2246	5.9	372.6
GanM	Yuzhong, Gansu	103.98	35.79	6	2762	7.2	374.5
GanZ	Ziwuling, Gansu	108.53	36.07	6	1296	8.5	556.5
IV	Liupanshan, Gansu	106.17	35.65	6	2336	6.3	554.5
AII	Ruoergai, Sichuang	102.71	34.11	7	3199	1.3	788.1
AIII	Ruoergai, Sichuang	102.47	33.40	5	3442	1.5	705.2
AI	Songpan, Sichuang	103.95	32.76	6	2763	9.9	709.4
SiS	Hongyuan, Sichuang	102.61	32.01	6	3328	8.4	797.5
SiMY	Li, Sichuang	102.68	31.80	6	3467	9.1	782
SiJC	Jinchuang, Sichuang	102.16	31.36	5	3367	12	704
SiD	Danba, Sichuang	101.67	30.61	6	3448	11.7	648.7
AVI	Kangding, Sichuang	101.22	30.04	6	3570	7.6	749.1
SiJ	Litang, Sichuang	100.39	29.75	7	3744	4.2	698.6
			Total	109			
*Hippophae rhamnoides* subsp. *yunnanensis*	Ganzi, Sichuang	100.27	28.63	3	3391		
*H. rhamnoides* subsp. *turkestanica*	Yili, Xinjiang	81.16	43.185	2	2006		
*Hippophae neurocarpa*	Hongyuan, Sichuang	102.40	32.46	3	3563		
*Hippophae tibetana*	Ruoergai, Sichuang	102.95	33.57	2	3452		
			Total	10			

To infer ancestral haplotypes and reconstruct the evolutionary history of Chinese seabuckthorn, we also collected the other two subspecies of *H. rhamnoides* (subsp. *yunnanensis* and subsp. *turkestanica*) and two related *Hippophae* species (*H. tibetana*, *Hippophae neurocarpa*) as out-groups.

DNA was extracted using the standard procedure of the Plant Genomic DNA kit (DP305; TIANGEN Biotech Co. Ltd., Beijing, China). Two chloroplast intergenic regions were amplified using *Trn*S-*Trn*G (Hamilton 1999) and *Trn*T-*Trn*Y (Shaw et al. 2005) primers. PCR was performed in 30 *μ*L reactions containing 10 mmol/L Tris–HCl (pH 8.3), 50 mmol/L KCl, 1.6 mmol/L MgCl_2_, 0.2 mmol/L each of four dNTPs, 0.75 U Taq polymerase (TaKaRa Company, Tokyo, Japan), 0.33 *μ*mol/L of each primer, and 40–60 ng of DNA templates. We amplified the intergenic fragments with the following procedure: 5 min at 95°C, followed by 30 cycles of denaturating for 1 min at 94°C, annealing for 50 sec at 55°C (for *Trn*S-*Trn*G) or 60°C (for *Trn*T-*Trn*Y), and extension for 1 min at 72°C, with a final extension step at 72°C for 8 min. The PCR products were sequenced at BGI LifeTech Co., Ltd., Beijing, China.

### Data analysis

The raw sequences were checked and aligned using CodonCode Aligner with clustal X module (http://www.codoncode.com/index.htm). The aligned results were rechecked by eye to correct some gaps that could be merged. Haplotypes were identified, and haplotype diversity was calculated using DNAsp 5.0 (Librado and Rozas 2009) by combining the results of *Trn*S-*Trn*G and *Trn*T-*Trn*Y. Indels were treated as a single site in the followed analysis. However, microsatellite polymorphism with poly structure was not considered in this study, for the divergent mutation mechanism between substitution and microsatellite. A median-joining haplotype network was constructed in NETWORK4.6 (http://www.fluxus-engineering.com/) under default settings. The spatial distribution of haplotypes was mapped by ArcMap 9.2 (ESRI Inc). All haplotypes have been deposited in both GenBank (Accession No.: JX311471–JX311481) and POPSET database (Accession No.: KM458855–KM458970).

The phylogenetic relationship among haplotypes was reconstructed using both Bayesian method in Beast 1.7.0 (Drummond et al. 2012) and maximum-likelihood method in MEGA5 (Tamura et al. 2011). All analyses used a substitution model HKY+G, selected by jModeltest (Darriba et al. 2012). In Beast, two independent run were undertaken by applying an uncorrelated lognormal relaxed clock (Drummond et al. 2002) and a Yule speciation process. For each MCMC run, 10 million generations were performed with sampling every 1000th generation, following a burn-in of the initial 10% cycles. MCMC samples were inspected in TRACER (version 1.5; Rambaut and Drummond 2009) to confirm sampling adequacy and convergence of the chains to a stationary distribution. Resulting chronograms were visualized in FIGTREE (version 1.3.1; http://tree.bio.ed.ac.uk/software/figtree/). In MEGA5, 1000 bootstraps were applied to infer the support rate of clades. Both methods used *H. tibetana* and *H. neurocarpa* as out-groups.

To uncover potential relationship between two closely related subspecies, *H. rhamnoides* subsp. *sinensis* and subsp. *yunnanensis*, we downloaded *Trn*S-*Trn*G data from paper Cheng et al. (2009) (GenBank Accession No.: EU100008–EU100010, HM769696). We constructed phylogenetic relationships of the two subspecies using the same two methods as above and the same out-groups selection (*H. tibetana* and *H. neurocarpa*).

**Figure 1:**
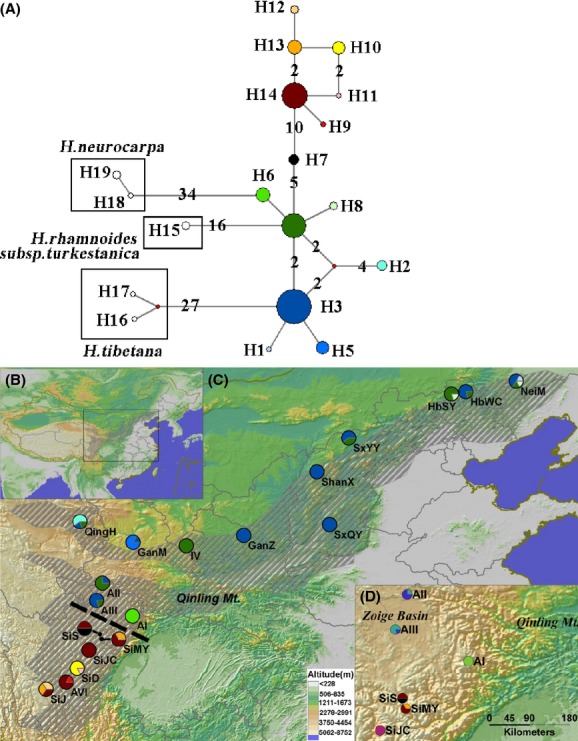
(A) A median joining network of 19 haplotypes found in our study. The circle size is proportional to the haplotype frequency. The number on the connection lines indicates the mutation steps between haplotypes, while for unmarked lines, the default number of mutation steps is one. (B) The subspecies range distribution over the study area. (C) The spatial distribution of haplotypes for Chinese seabuckthorn. The haplotype colors are identical in (A and C). The shaded area is the range distribution of the subspecies according to Lian and Chen (1991). The black dashed line indicates the transition area where the haplotype group changes from the North type (above the line) to the Southwest type (below the line). (D) A detail map around the transition region in (C).

A mismatch distribution analysis was conducted using Alequin 3.01 (Excoffier and Schneider 2005) to test whether the populations had experienced expansion. As the North and Southwest regions harbored distinctive haplotypes (Fig. 1), we conducted the mismatch analysis separately for the North and Southwest populations. We used two methods to evaluate whether populations had experienced a sudden expansion: (1) sum of squared deviation (SSD); and (2) Harpending's raggedness index (HRag). To test the significance of the observed date from simulated data with sudden expansion, 100 bootstrap replicates were applied. If the sudden expansion model was not rejected, then an expansion parameter (*τ*) could be calculated from the analysis, which equals the product of expansion time since it started (T) and the mutation rate for the whole sequences per generation (u): *τ* = 2uT. The mutation rate, u, can be calculated by the following equation: u = *μ*k, where *μ* is the substitution rate per site and k is the average length of analyzed sequences (1496 bp in this study). Therefore, we could estimate the expansion time since it started for both population groups. When studying *H. tibetana*, Wang et al. (2010) employed a substitution rate 4.87 × 10^−10^ substitutions per site per year (s/s/y) for one intergenic region of chloroplast (*Trn*T-*Trn*F) and found good consistency of the divergence time among chloroplast, ITS, and fossil datasets. Hence, we used this substitution rate in our study to estimate the expansion time (T) of the North populations and the Southwest populations.

The hierarchical partitioning of genetic diversity among identified groups of populations was calculated with analyses of molecular variance (AMOVA) in the program ARLEQUIN 3.01 (Excoffier et al. 2006).

## Results

The aligned sequences of *Trn*S-*Trn*G and *Trn*T-*Trn*Y were 1496 bp (629 bp and 867 bp, respectively). For all 119 individuals, 71 parsimony informative sites were found and 20 were unique for Chinese seabuckthorn. Large indels existed in both intergenic regions. *Trn*S-*Trn*G had three common indels, ranging from 2 bp to 123 bp. One indel (2 bp) was unique for *H. tibetana*. *Trn*T-*Trn*Y had two common indels, ranging from 7 bp to 103 bp. One indel (7 bp)was unique for *H. neurocarpa*. The length of both large indels (>100 bp) varied among species and also within the subspecies.

We observed a total of 19 haplotypes by combining the two intergenic regions. Chinese seabuckthorn had 12 unique haplotypes, while *H. rhamnoides* subsp. *yunnanensis* had no unique haplotypes in our samples, but rather two haplotypes shared with Chinese seabuckthorn (H13, H14). No shared haplotypes were found among Chinese seabuckthorn and the other three species (subspecies). The haplotype diversity of Chinese seabuckthorn was 0.691 ± 0.042.

**Figure 2:**
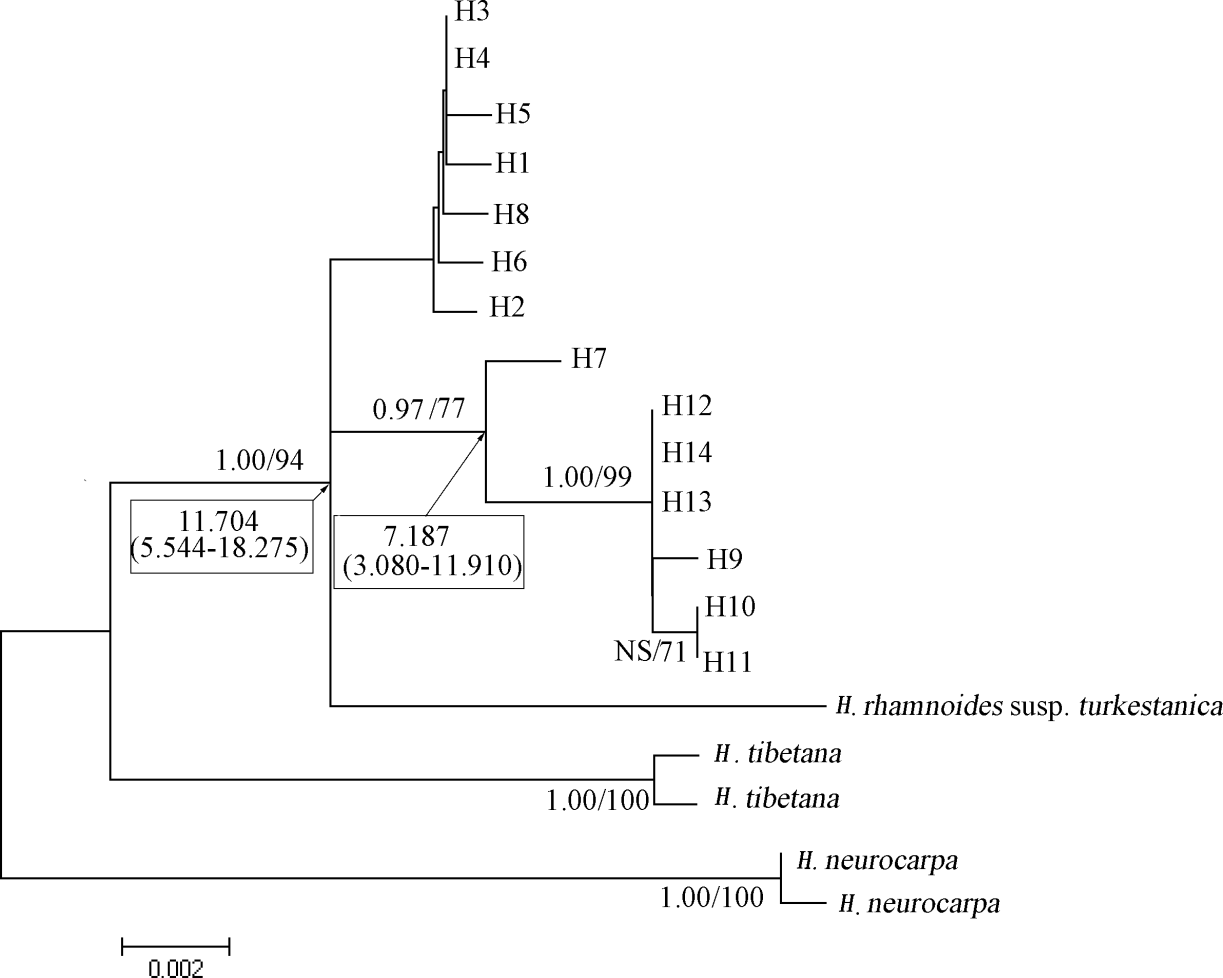
The maximum-likelihood tree topology of the cpDNA haplotypes detected from the *Trn*S-*Trn*G and *Trn*T-*Trn*Y region of Chinese seabuckthorn; numbers above the branches indicate Bayesian posterior probabilities (left) and the bootstrap values for ML(right) analyses, respectively; inferred dates in Ma before present (95% confidence interval) are given in the rectangular boxes. NS, nonsupported.

We found two major haplotype groups of Chinese seabuckthorn in our study, which were separated by 15 mutation steps (Fig. 1A) and supported by bayesian and ML trees (Fig. 2). These two groups were distributed in distinct spatial areas without any overlap (Fig. 1B). The warm colored haplotypes (red and yellow in Fig. 1) were limited to the Southwest corner of the subspecies range (herein referred to as “Southwest populations”), while the cold colored haplotypes (blue and green) were widely distributed in the North range (herein referred to as “North populations”), including the high elevation regions on the QTP and the lower altitude area in the Northeast range. Haplotype H14 was common in the Southwest populations, while H3 and H4 were common in the North populations. The populations on the QTP harbored far more private haplotypes than the lower land (only two: H1 and H8). AMOVA indicated 89.96% of the genetic variation existing among the two groups (Table 3).

**Figure 3:**
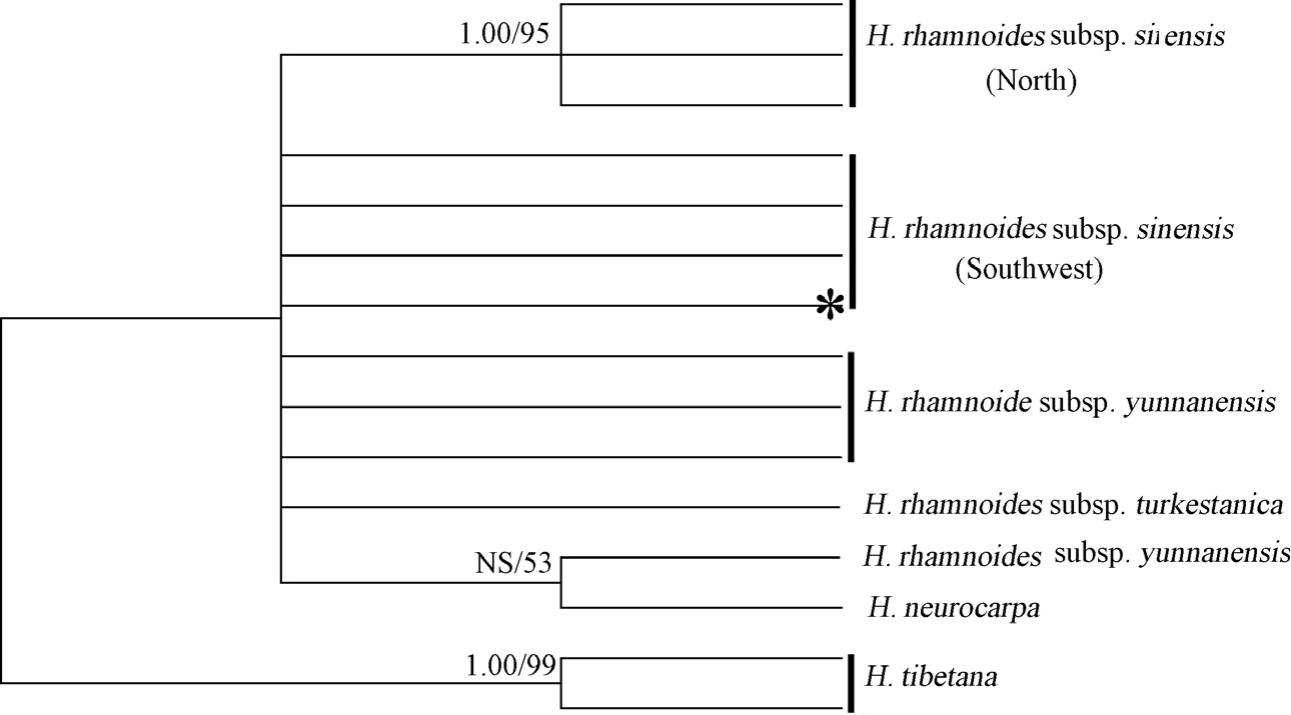
Comparison of *Trn*S-*Trn*G results in this study with published data of *Hippophae rhamnoides* subsp. *yunnanensis* (Cheng et al. 2009). ML tree was constructed in Mega5. The values adjacent to the branches indicate the support value based on 2000 bootstrap replications. Only bootstrap values larger than 50% are shown. The node marked with a star indicates the haplotypes of Chinese seabuckthorn that are shared with *H. rhamnoides* subsp. *yunnanensis*. NS, nonsupported.

The coalescent time of Chinese seabuckthorn was estimated around 11.704 (5.544–18.275) million years ago, and the coalescent time of Southwest populations was 7.187 (3.080–11.910) million years ago (Fig. 2). The North populations started expansion around the early of Pleistocene 3.025 (0.868–5.770) million years ago, and the Southwest populations started since 1.736 (0.485–4.151) million years ago (Table 2).

**Table 2:** Results of mismatch analysis for the Southwest and North range of Chinese seabuckthorn, respectively. Goodness of fit under a sudden expansion model was tested using the sum of squared deviations (SSD) and Harpending's raggedness index (HRag). Expansion parameters (*τ*) and expansion time (*T*) since it started were estimated using a substitution rate of 4.87 × 10^−10^ substitutions per site per year (s/s/y)

Populations	*τ* (95% intervals)	SSD (*P*-value)	HRag (*P*-value)	*T* (million years ago)
Southwest	4.408 (1.265, 8.407)	0.052 (0.36)	0.135 (0.36)	3.025 (0.868–5.770)
North	2.53 (0.707, 6.048)	0.024 (0.27)	0.073 (0.30)	1.736 (0.485–4.151)

## Discussion

Based on chloroplast fragment sequencing, our study revealed relatively high diversity in Chinese seabuckthorn. It had 14 haplotypes, containing 28 polymorphic sites, with most of the haplotypes distributing on the QTP. The genetic diversity level was comparable with other *Hippophae* species. Comparison studies between two species, *Hippophae gyantsensis* and *H. rhamnoides* subsp. *yunnanensis*, on QTP found 11 haplotypes with 49 polymorphic sites (Cheng et al. 2009). Another *Hippophae* species *H. tibetana* yielded extreme high diversity (50 haplotypes with 31 polymorphic sites; Wang et al. 2010). However, considering the *H. tibetana* study had much more extensive sampling (37 populations, 891 individuals) than the other studies, we believed the diversity of *Hippophae* species or subspecies around QTP was generally similar, reflecting similar demographic history. In addition, a deep phylogenetic relationship was found between Chinese seabuckthorn and *H. rhamnoides* subsp. *turkestanica* (15 mutation steps, Fig. 1), which may suggest a long independent evolution history of these subspecies. Alike pattern was recovered in another four European subspecies of *H. rhamnoides*, where each subspecies harbored unique diversity from others (Bartish et al. 2006). Hence, the diversity resource of *H. rhamnoides* was extremely high and patchy-distributed throughout its species range.

We found two sharing haplotypes between Chinese seabuckthorn and *H. rhamnoides* subsp. *yunnanensis*. The sharing haplotypes occurred only in Southwest populations, where the two subspecies range overlapped. Moreover, phylogeny studies based on morphological and molecular data both supported a close relationship between these two subspecies (Rousi 1971; Lian et al. 1997c; Bartish et al. 2002). Hence, it is possible that these two sharing haplotypes may originate from introgression between subspecies. However, by combining *H. rhamnoides* subsp. *yunnanensis* data from Cheng et al. (2009), we found haplotypes of *Trn*S-*Trn*G fragment of both subspecies were mixed in the Bayesian or ML tree (Fig. 3). Two haplotypes of subsp. *yunnanensis* clustered with haplotypes from North populations of Chinese seabuckthorn, while another two haplotypes clustered with ones from Southwest populations. Therefore, although introgression may be common between subspecies of *H. rhamnoides* or between species of genus *Hippophae* (e.g., Cheng et al. 2009), the sharing haplotypes in our study and mixture of haplotypes in the phylogenetic trees more likely suggested incomplete lineage sorting pattern between the two subspecies. The insight of incomplete lineage sorting can also be supported by the recent divergence of the two subspecies, as they had very close relationship.

In our study, two distinct, spatially isolated haplotype groups with up to 15 mutation steps were found (Fig. 1, Table 3). Both groups were distributed on the QTP, while only one haplotype group spread to the range with low elevation in the East. The haplotype distribution pattern is similar with species that coexist on the QTP and North China. For example, an alpine shrub, *Potentilla fruticosa*, revealed higher diversity and more ancestral haplotypes on the QTP than the adjacent area (Li et al. 2010). Another shrub species, *Ostryopsis davidiana*, has a more similar pattern with Chinese seabuckthorn, with a haplotype transition area from the south type to the north type located in the Qinling Mountain (Tian et al. 2009). In our study, we found the haplotype transition area of Chinese seabuckthorn was in the south of Zoige Basin, which has a close tectonic relationship with the Qinling Mountain (Gao et al. 2006). This consistency among species reflects a common history that species have experienced during the climatic and geological changes on the QTP and its adjacent area.

**Table 3:** Analyses of molecular variance (AMOVA) based on chloroplast haplotype frequencies for North and Southwest populations of Chinese seabuckthorn

Source of variation	df	Percentage of total variance (%)
Among groups	1	89.96
Among populations within groups	16	4.52
Within populations	86	5.52

All levels of variation were significant.

The two observed haplotype groups in our study have an ancient divergence time, between Miocene and Pliocene, and an subsequence expansion during the early of Pleistocene (Fig. 2, Table 2). They have little overlap in space, and the transition area between these two groups is sharp, <200 km. This suggests that their seed dispersal is limited to these two regions. Chinese seabuckthorn disperse seeds mainly via birds, and it is possible that barriers, like high mountains, may prevent birds from moving from one site to another. Zhang et al. (2006) found a significant genetic differentiation between Chinese seabuckthorn populations located on the two sides of Qilian Mountain, the elevation of which is higher than 4000 m. However, we noticed that most of the transition area was located in a basin with elevation <4000 m, while the mountains between populations in the Southwest region are mostly higher than 4000 m. If the high mountains were the true barrier, then we should observe more private haplotypes in the Southwest region. Moreover, morphology studies indicated morphological divergence may exist between regions. The fruit in the Southwest region was mostly big and orange, with a pearlike shape, while in the North region, the fruit is smaller and red, with an applelike shape (Lian et al. 1997b). The habitats between these two regions were also differentiated mostly in altitude and precipitation (one-way ANOVA test; altitude: *P* = 0.001; annual precipitation: *P* = 0.003; annual mean temperature: *P* = 0.052). High elevation may increase precipitation and the two factors interact with each other. Collectively, these divergences between regions in habitat, genetics, and morphology suggested long independent evolution history between regions. Potential adaptation between regions may occur but still need more genome and morphology proofs in future.

Mismatch distributions of the North and the Southwest populations indicated a significant expansion occurring 3.025 and 1.736 million years ago, respectively (Table 2). The expansion time is comparable with some species on the QTP. Three lineages of *H. tibetana* started expansion around 1.257–3.625 million years ago, while the whole populations might started around 13.740 million years ago (the expansion time of *H. tibetana* was recalculated using the methods described in this paper; Wang et al. 2010). However, some grass species (*Pedicularis longiflora*) revealed a younger expansion time in some localities on the QTP, <0.3 million years ago (Yang et al. 2008). For the other subspecies of *H. rhamnoides* in Europe, the expansion was more recent, with most expansion starting after the last glacial maximum (LGM, 0.02 million years ago; Bartish et al. 2006). Two reasons may contribute to this difference in population growth between the subspecies of *H. rhamnoides*. Firstly, large ice sheets covered most of the European area during the glacial period, while in China, only mountain areas were glaciated and there was no clear proof of land ice sheets (Shi et al. 2006). Hence, not until after the LGM when the large land ice sheets had disappeared would very limited expansions occur in Europe. This point was also supported by patterns of other species (Sharbel et al. 2000; Petit et al. 2002; Grivet et al. 2006). Secondly, the vast heterogeneous landscape on the QTP provides an opportunity to decouple the local climate from global change (Dobrowski 2011; Hampe and Jump 2011), allowing extensive microrefugia to exist on the plateau. This has been highly supported by pollen records (Tang and Shen 1996; Tang et al. 2000) and phylogeography studies of many QTP species (such as Yang et al. 2008; Li et al. 2010; Wang et al. 2010). In our study, we found that some private haplotypes reached very high diversity or were fixed in some populations, such as H7 in population SiS, H10 in population SiD, H6 in population AI, and H2 in population QingH (Fig. 1). These localities on the QTP had high potential to be microrefugia for the species to survival the glaciation period. Therefore, unlike the subspecies of *H. rhamnoides* in Europe, Chinese seabuckthorn was able to survive the glacial period, expand during Pleistocene, and ultimately sustain higher genetic diversity.

## Conclusion

Our phylogeography study of Chinese seabuckthorn demonstrates that historical climate change and local terrain conditions play important roles in determining modern spatial genetic patterns. The genetic diversity of Chinese seabuckthorn was comparable with other *Hippophae* species or subspecies on the QTP. Two distinct haplotype groups with >15 mutation step difference were found to be separately located in the Southwest and the North region. Both haplotype groups started expansion at the end of Neogene and the early period of Pleistocene, with the North populations being more ancient. Additional morphological difference among groups suggests a long independent colonization history of these two regions. Multiple populations on the QTP harbored private haplotypes with high frequency, suggesting multiple microrefugia existed during the glaciation period. Lack of extensive land ice sheets and the heterogeneous landscape within the range of Chinese seabuckthorn, especially on the QTP, provided the preconditions for microrefugia. Additionally, we found an incomplete lineage sorting pattern between Chinese seabuckthorn and *H. rhamnoides* subsp. *yunnanensis*, which supported the close relationship between the two subspecies. We also found a large discrepancy between our study subspecies and subsp. *turkestanica*. As the truth that most of the subspecies harbored specific diversity (Bartish et al. 2006), *H. rhamnoides* should have sustained abundant genetic resources throughout its species range, which could be useful for its future cultivation.
